# An efficient pyrrolysyl-tRNA synthetase for economical production of MeHis-containing enzymes†

**DOI:** 10.1039/d4fd00019f

**Published:** 2024-03-07

**Authors:** Amy E. Hutton, Jake Foster, James E. J. Sanders, Christopher J. Taylor, Stefan A. Hoffmann, Yizhi Cai, Sarah L. Lovelock, Anthony P. Green

**Affiliations:** a Manchester Institute of Biotechnology, School of Chemistry, The University of Manchester Manchester UK Anthony.green@manchester.ac.uk Sarah.lovelock@manchester.ac.uk

## Abstract

Genetic code expansion has emerged as a powerful tool in enzyme design and engineering, providing new insights into sophisticated catalytic mechanisms and enabling the development of enzymes with new catalytic functions. In this regard, the non-canonical histidine analogue *N*_δ_-methylhistidine (MeHis) has proven especially versatile due to its ability to serve as a metal coordinating ligand or a catalytic nucleophile with a similar mode of reactivity to small molecule catalysts such as 4-dimethylaminopyridine (DMAP). Here we report the development of a highly efficient aminoacyl tRNA synthetase (G1PylRS^MIFAF^) for encoding MeHis into proteins, by transplanting five known active site mutations from *Methanomethylophilus alvus* (*Ma*PylRS) into the single domain PylRS from *Methanogenic archaeon* ISO4-G1. In contrast to the high concentrations of MeHis (5–10 mM) needed with the *Ma* system, G1PylRS^MIFAF^ can operate efficiently using MeHis concentrations of ∼0.1 mM, allowing more economical production of a range of MeHis-containing enzymes in high titres. Interestingly G1PylRS^MIFAF^ is also a ‘polyspecific’ aminoacyl tRNA synthetase (aaRS), enabling incorporation of five different non-canonical amino acids (ncAAs) including 3-pyridylalanine and 2-fluorophenylalanine. This study provides an important step towards scalable production of engineered enzymes that contain non-canonical amino acids such as MeHis as key catalytic elements.

## Abstract 2

A highly efficient aminoacyl tRNA synthetase (G1PylRS^MIFAF^) has been developed to produce MeHis-containing proteins. High protein titres can be achieved with low ncAA concentrations (0.1 mM) enabling more economical production of MeHis-containing enzymes.

## Graphical abstract

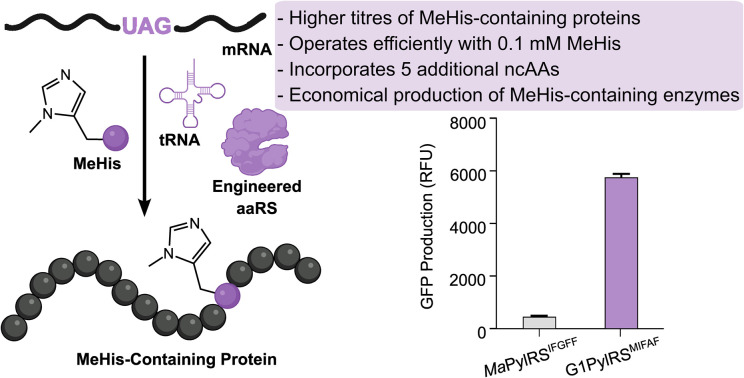

## Introduction

In nature proteins perform a vast array of functions, including accelerating biochemical reactions, transporting molecules across membranes, providing structural support, and controlling signalling processes. Recent advances in high-throughput computation and experimentation have given us unprecedented control over protein sequence, structure and function, resulting in the development of a diverse array of engineered protein therapeutics, biocatalysts, and advanced biomaterials.^1–6^ At present, our approaches to protein design, engineering and production typically only make use of nature's standard alphabet of twenty canonical amino acid building blocks. These standard amino acids are limited in their chemical diversity, which ultimately restricts our ability to develop proteins with new functions and desirable properties. To address this fundamental limitation, genetic code expansion (GCE) has emerged as a powerful and versatile technology to site selectively install new functional elements into proteins as non-canonical amino acids (ncAAs).^7,8^ GCE employs orthogonal aminoacyl tRNA synthetase (aaRS)/tRNA pairs to direct the incorporation of ncAAs in response to a reassigned codon (most commonly the amber codon UAG) introduced into a gene of interest. To date, a variety of aaRS/tRNA pairs have been developed which display the required orthogonality across a range of host organisms. Pyrrolysyl aaRS/^Pyl^tRNA_CUA_ pairs from methanogenic archaea have proven especially versatile, having been re-engineered to encode several hundred ncAAs in bacteria, yeast and mammalian cell lines.^9^ These systems have allowed the development of new protein therapeutics, precision bioconjugates, responsive materials, protein-based vaccines and new biocontainment strategies.

**Fig. 1:**
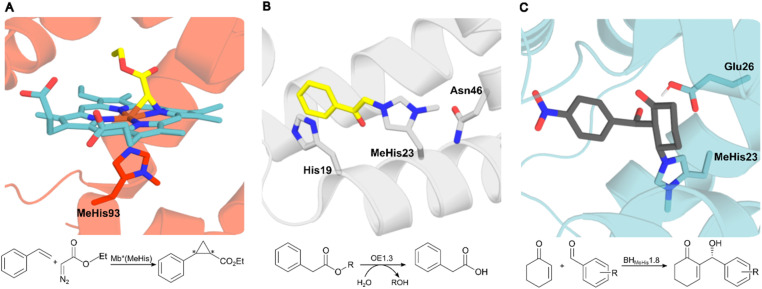
Crystal structures of MeHis-containing enzymes. (A) The crystal structure of the myoglobin variant Mb*(MeHis) (PDB: 6G5B), in which substitution of His93 with MeHis affords an oxygen tolerant carbene transferase.^23^ MeHis is shown as atom-coloured sticks, carbon in red, showing the Fe(iii) carbenoid complex (atom-coloured stick, carbons in blue and yellow). (B) The designed esterase OE1.3 uses MeHis23 as a catalytic nucleophile.^15^ The crystal structure (PDB: 6Q7R) shows MeHis23 (atom-coloured sticks, carbons in grey) alkylated with the mechanistic inhibitor bromoacetophenone (atom-coloured sticks, carbons in yellow). (C) Snapshot from an MD simulation of BH_MeHis_1.8_Int2 complex. BH_MeHis_1.8 uses MeHis23 as a catalytic nucleophile (PDB: 8BP0, atom-coloured sticks, carbons in blue). Int2 is shown in atom-coloured sticks with carbons in grey.^20^

The availability of an expanded genetic code also opens exciting new opportunities in enzyme design and engineering. For example, GCE has been used to improve enzyme activity and stability,^10,11^ to probe complex biological mechanisms,^12–14^ and to develop enzymes with functions and modes of catalysis beyond those found in nature.^15–20^ The non-canonical histidine analogue, *N*_δ_-methylhistidine (MeHis), has proven to be an especially versatile tool in enzyme design and engineering research, leading to catalytically modified enzymes with augmented properties and entirely new functions, as well as new biocontainment strategies (Fig. 1).^15,20,21^ For example, MeHis has been used as a metal chelating ligand to probe the mechanisms of heme enzymes including ascorbate peroxidase and cytochrome *c* peroxidase.^10,12^ MeHis ligands have also been used to augment metalloenzyme function, leading to improvements in both peroxidase and carbene transferase activities in engineered myoglobin variants.^11,22,23^ MeHis has also been shown to act as a potent catalytic nucleophile, leading to the development of artificial hydrolases and proficient enzymes for valuable non-biological transformations such as the Morita–Baylis–Hillman reaction.^15,20^

To capitalize on these recent advances, it is important that we are able to produce MeHis-containing enzymes in an efficient and economical manner. At present, the engineered aaRS/tRNA pairs used to encode MeHis are relatively inefficient, typically requiring 5–10 mM concentrations of the expensive ncAA to be supplemented to the culture medium. For context, for a 30 kDa protein produced at 100 mg per litre of culture, this equates to only 0.03–0.06% of MeHis being incorporated into the target protein. Furthermore, even at these high MeHis concentrations, a substantial proportion of undesired truncated protein is typically observed. It is therefore evident that for any future large-scale applications of MeHis-containing enzymes, the efficiency of modified protein production will have to be significantly improved. Here we report an efficient system for producing MeHis-containing proteins in high titres using only low concentrations of ncAA, providing an important step towards scalable production of these modified biocatalysts.

## Results and discussion

**Fig. 2:**
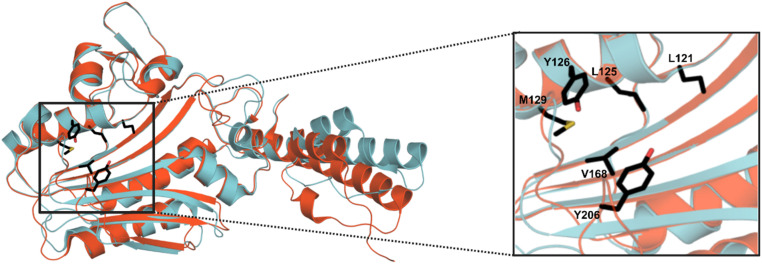
Crystal structure overlay of *Ma*PylRS and G1PylRS. An overlay of wild-type *Ma*PylRS (red, PDB: 6JP2)^26^ and wild-type G1PylRS (blue, PDB: 8IFJ).^29^ Two distinct sets of mutations have been reported to confer activity towards MeHis incorporation in *Ma*PylRS. The residues mutated in these two variants are shown as atom-coloured sticks with black carbons. The specific mutations present in each variant are detailed in the main body of text.

MeHis is commonly incorporated into target proteins using engineered PylRS homologs from *Methanosarcina mazei* (*Mm*), *Methanosarcina barkeri* (*Mb*) or *Methanomethylophilus alvus* (*Ma*).^24,25^ Two distinct sets of mutations have been reported to confer activity towards MeHis incorporation, either L121M, L125I, Y126F, M129A and V168F or L125I, Y126F, M129G, V168F and Y206F (based on *Ma* numbering), affording *Ma*PylRS^MIFAF^ or *Ma*PylRS^IFGFF^ respectively (Fig. 2).^26–28^ A recent study showed that *Ma*PylRS^IFGFF^ gave modest improvements in protein yield compared with the analogous *Mm*PylRS^IFGFF^ variant, although high concentrations of MeHis are required in both cases. With the aim of identifying more efficient systems, we elected to explore a wider range of PylRS homologs, namely *Methanogenic archaeon* ISO4-G1 (G1) PylRS and *Methanomassiliicoccales archaeon* RumEn M1 (RumEn) PylRS.^25^ Similar to *Ma*PylRS, these homologs lack the N-terminal tRNA binding domain that is essential for activity in *Mm*PylRS and *Mb*PylRS. To develop engineered G1PylRS and RumEnPylRS for encoding MeHis, we mapped mutations from *Ma*PylRS^MIFAF^ and *Ma*PylRS^IFGFF^ into these homologs, which along with their respective cognate ^Pyl^tRNA_CUA_, gave rise to four new aaRS/tRNA pairs for experimental characterization.

**Fig. 3:**
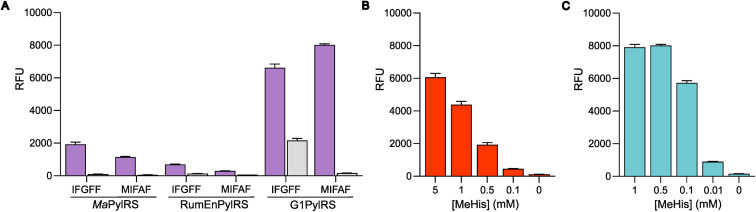
Production of MeHis-containing GFP using engineered PylRS homologs. (A) Bar chart showing production of GFP containing MeHis at position 150, using either *Ma*PylRS^MIFAF/IFGFF^/^Pyl^tRNA_CUA_, Rum*En*PylRS^MIFAF/IFGFF^/^Pyl^tRNA_CUA_ or G1PylRS^MIFAF/IFGFF^/^Pyl^tRNA_CUA_ pairs. Cultures grown in the presence of 0.5 mM MeHis (purple bars) or with no MeHis supplemented (grey bars). Error bars represent the standard deviation of measurements made in triplicate. (B) Bar chart showing GFP production in cultures containing varying MeHis concentrations (0–5 mM) using *Ma*PylRS^IFGFF^/^Pyl^tRNA_CUA_. Error bars represent the standard deviation of measurements made in triplicate. (C) Bar chart showing GFP production in cultures containing varying MeHis concentrations (0–1 mM) using G1PylRS^MIFAF^/^Pyl^tRNA_CUA_. Error bars represent the standard deviation of measurements made in triplicate.

The resulting G1PylRS^MIFAF/IFGFF^ and RumEnPylRS^MIFAF/IFGFF^ variants were evaluated using an established GFP production assay and their activity compared to the analogous *Ma* systems. Of the two existing *Ma* variants, *Ma*PylRS^IFGFF^ was shown to be slightly more effective in suppressing the UAG codon to produce full length GFP containing MeHis at position 150 (Fig. 3A). The UAG suppression efficiency of RumEnPylRS^MIFAF/IFGFF^ was substantially reduced compared to the *Ma* variants. In contrast, both active-site transplanted G1PylRS variants showed substantially improved UAG suppression efficiency. G1PylRS^MIFAF^ displays both high activity and specificity for MeHis (purple bars) over incorporation of canonical amino acids (grey bars), whereas the G1PylRS^IFGFF^ variant suffers from a high background of phenylalanine incorporation in cultures grown in the absence of MeHis. This newly engineered G1PylRS^MIFAF^ variant produces approximately 4-fold more full length GFP than *Ma*PylRS^IFGFF^ when 0.5 mM MeHis is supplied to the culture medium.

**Fig. 4:**
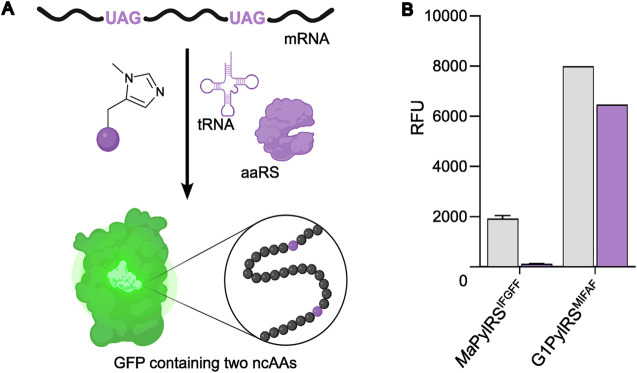
Production of GFP containing two MeHis residues. (A) Schematic representation of modified GFP expression, introducing two MeHis residues in response to UAG codons at positions 40 and 150. (B) Bar chart showing GFP production containing MeHis at either position 150 (grey bars) or at positions 40 and 150 (purple bars) using either *Ma*PylRS^IFGFF^/^Pyl^tRNA_CUA_ or G1PylRS^MIFAF^/^Pyl^tRNA_CUA_. Cultures were supplemented with 0.5 mM MeHis. Error bars represent the standard deviation of measurements made in triplicate.

To further compare the G1PylRS^MIFAF^ and *Ma*PylRS^IFGFF^ systems, we next evaluated GFP production across a range of MeHis concentrations (Fig. 3B, C and ESI Fig. S1†). Remarkably, G1PylRS^MIFAF^ can operate efficiently using a MeHis concentration of 0.1 mM, with detectable levels of GFP production even observed at 0.01 mM. Increasing the MeHis concentration to 0.5 mM or 1 mM led to only modest improvements in MeHis incorporation, suggesting that G1PylRS^MIFAF^ is saturated at a concentration between 0.1 and 0.5 mM. For comparison, G1PylRS^MIFAF^ was a more effective aaRS at 0.1 mM MeHis than *Ma*PylRS^IFGFF^ at 1 mM. Even using 50 times more MeHis (5 mM), *Ma*PylRS^IFGFF^ is only marginally more active. To illustrate the efficacy of G1PylRS^MIFAF^, we further increased the stringency of GFP production assays by introducing an additional UAG codon at position 40 (Fig. 4). The G1PylRS^MIFAF^ system is able to efficiently read through two UAG codons to produce GFP containing MeHis at positions 40 and 150, with only minor reductions in protein yield (Fig. 4B). In contrast, with the less efficient *Ma*PylRS^IFGFF^ system yields of doubly modified GFP are extremely low. It is notable that many enzymes contain multiple catalytically important histidine residues.^30–33^ The ability to efficiently produce proteins containing multiple MeHis residues will open up new opportunities to study and/or tune the functions of these enzymes.

Having established an efficient aaRS/tRNA pair, our attention now turned to the production of engineered enzymes that use MeHis as an important catalytic element. To this end we selected the engineered peroxidase APX2_MeHis, where MeHis serves as an axial ligand and leads to dramatically improved turnover numbers, and designed enzymes OE1.4 (stereoselective hydrolase) and BH_MeHis_1.8 (Morita–Baylis–Hillmanase) that both employ MeHis as a catalytic nucleophile.^10,15,20^ Using only 0.1 mM MeHis, these engineered enzymes are all produced in >100 mg L^−1^ in standard laboratory *Escherichia coli* strains and culture conditions, corresponding to an impressive 4–6% of the total MeHis supplemented being incorporated into protein (Table 1). In all cases, protein yields achieved with G1PylRS^MIFAF^ are substantially higher than those produced with *Ma*PylRS^IFGFF^ using 10 times higher MeHis concentrations (1 mM).

**Table 1:** Protein titres of engineered enzymes containing MeHis

Synthetase	[MeHis] (mM)	Protein yield (mg L^−1^)		
		APX2	BH_MeHis_1.8	OE1.4
*Ma*PylRS^IFGFF^	1	107	58	71
G1PylRS^MIFAF^	0.1	188	127	106

**Fig. 5:**
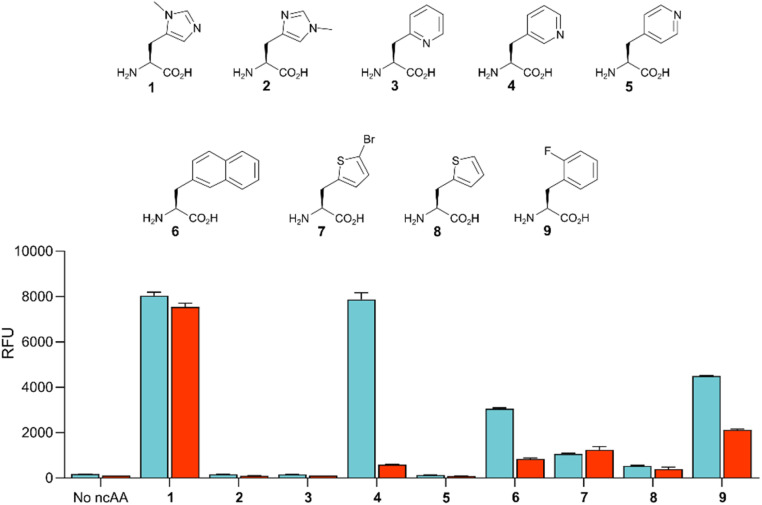
Comparison of the polyspecificity of G1PylRS^MIFAF^ and *Ma*PylRS^IFGFF^ systems. Bar chart showing the activities of G1PylRS^MIFAF^ (blue) and *Ma*PylRS^IFGFF^ (red) towards a small panel of ncAAs (10 mM, 1–9). PylRS activity was determined by monitoring production of modified GFP containing the appropriate ncAA at position 150. Error bars represent the standard deviation of measurements made in triplicate.

Given the high efficiency of G1PylRS^MIFAF^, we wondered whether this aaRS is highly specific for MeHis or whether it could also be used to encode other ncAAs. We therefore tested G1PylRS^MIFAF^ activity towards a small panel of ncAAs (10 mM) using the aforementioned GFP production assay. In addition to MeHis, G1PylRS^MIFAF^ is able to encode five of the other ncAAs tested (Fig. 5, structures 4, 6, 7, 8, and 9). These substrates include the hydrophobic ncAAs 3-(2-naphthyl)alanine and 2-fluorophenylalanine, as well as 3-pyridylalanine, a potentially valuable histidine analogue that is a poor substrate for *Ma*PylRS^IFGFF^ and *Ma*PylRS^MIFAF^ (ESI Fig. S2†). The ability of G1PylRS^MIFAF^ to efficiently discriminate between phenylalanine and 2-fluorophenylalanine is particularly notable.

## Conclusion

In summary, we have developed a highly efficient aaRS for encoding MeHis by introducing five known active site mutations into a single domain PylRS from *Methanogenic archaeon* ISO4-G1. The successful development of G1PylRS^MIFAF^ serves to highlight the importance of exploring a wider range of PylRS homologs when developing orthogonal translation components. This G1PylRS^MIFAF^ has allowed the efficient and economical production of a range of MeHis-containing enzymes using only 0.1 mM MeHis supplemented to the culture medium. Moving forward, there are several avenues for investigation to further enhance the production of valuable proteins containing MeHis to underpin any future commercial applications. Firstly, it is likely that even more efficient G1PylRS^MIFAF^ descendants can be developed through directed evolution using established high-throughput assays. Switching to high-density fermentation technologies will also likely boost protein yields. Secondly, we can take advantage of engineered or synthetic *E. coli* strains that have been specifically tailored for more efficient ncAA incorporation.^8,34–36^ Alternatively, we can explore engineered yeast strains or mammalian cell lines that can be advantageous for selected protein applications.^37–39^ Finally, considering MeHis is a naturally occurring amino acid, we can envision the development of engineered production hosts that contain the necessary biosynthetic machinery to produce MeHis and direct its selected incorporation into target proteins. For these reasons, we are optimistic that the work presented in this paper will provide an important step towards commercially viable production of MeHis-containing enzymes.

## Methods

### Materials

All chemicals and biological materials were obtained from commercial suppliers. *Escherichia coli* DH10β cells were purchased from Thermo Fisher. *Escherichia coli* BL21(DE3), Q5 polymerase, T4 ligase and restriction enzymes from New England BioLabs (NEB). PylRS and tRNA gene sequences were synthesized by Twist Bioscience. Primers were synthesized by IDT. Kanamycin, chloramphenicol and 5-aminolevulinic acid were purchased from Sigma-Aldrich. (*S*)-2-Amino-3-(5-bromothiophen-2-yl) propanoic acid, 2-fluoro-l-phenylalanine, 3-(3-pyridyl)-l-alanine and 3-(2-naphthyl)-l-alanine were purchased from Fluorochem. 3-(2-Thienyl)-l-alanine and 3-(2-pyridyl)-l-alanine were purchased from Fisher Scientific UK. 3-(4-Pyridyl)-l-alanine was purchased from Alfa-Aesar. H-His(1-Me)-OH was from abcr. LB agar, LB media, 2xYT media, isopropyl-β-d-1-thiogalactopyranside (IPTG) and arabinose were from Formedium. H-His(3-Me)-OH (MeHis) was from Bachem.

Defined autoinducing medium (500 mL) had the following composition: 25 mL aspartate (5%, pH = 7.5), 25 mL glycerol (10% w/v), 20 mL 18-amino-acid mix (5 g L^−1^ glutamic acid, 5 g L^−1^ aspartic acid, 5 g L^−1^ lysine·HCl, 5 g L^−1^ arginine·HCl, 5 g L^−1^ alanine, 5 g L^−1^ proline, 5 g L^−1^ glycine, 5 g L^−1^ threonine, 5 g L^−1^ serine, 5 g L^−1^ glutamine, 5 g L^−1^ asparagine·H_2_O, 5 g L^−1^ valine, 5 g L^−1^ leucine, 5 g L^−1^ isoleucine, 5 g L^−1^ phenylalanine, 5 g L^−1^ tryptophan, 5 g L^−1^ methionine, histidine-HCl-H_2_O 5 g L^−1^, sterile filtered), 1.25 mL arabinose (20% w/v), 20 mL 25 × M salts (0.625 M NaH_2_PO_4_, 0.625 M KH_2_PO_4_, 1.25 M NH_4_Cl, 0.125 M Na_2_SO_4_), 1 mL MgSO_4_ (1 M), 0.625 mL glucose (40% w/v), 100 μL 5000 × trace metals solution (20 mM CaCl_2_·2H_2_O, 10 mM MnCl_2_·H_2_O, 10 mM ZnSO_4_·7H_2_O, 2 mM CoCl_2_·6H_2_O, 2 mM CuCl_2_, 2 mM NiCl_2_, 2 mM Na_2_MoO_4_·2H_2_O, 2 mM NaSeO_3_, 2 mM H_3_BO_3_, 50 mM FeCl_3_), 1 mL MgSO_4_ (1 M), 500 μL IPTG (0.1 M). The solution was made up with sterile water to 500 mL.

### DNA constructs

pEVOL_*Ma*PylRS^IFGFF^/*Ma*^Pyl^tRNA_CUA_ was available from a previous study.^26^ PylRS genes (G1PylRS^MIFAF^ and RumEnPylRS^MIFAF^), optimized for *E. coli* expression, and the tRNAs (G1^pyl^tRNA_CUA_ and RumEn^pyl^tRNA_CUA_) were synthesized by Twist Bioscience. Two copies of each PylRS gene and their corresponding tRNA were cloned into their respective pEVOL vectors using *NdeI*/*PstI* and *BglII*/*SalI* restriction sites for PylRS genes and *ApaLI*/*XhoI* for the tRNA. To make *Ma*PylRS^MIFAF^, G1PylRS^IFGFF^ and RumEnPylRS^IFGFF^ primers to introduce the required mutations were used to make gene fragments which were combined using overlap extension PCR. Two copies of each gene were cloned into their respective pEVOL vectors using *NdeI*/*PstI* and *BglII*/*SalI* restriction sites.

### GFP expression assays

Chemically competent *E. coli* BL21(DE3) cells containing the appropriate pEVOL vector were transformed with either pET28_GFP_Asn150TAG or pET28_GFP_Asn40TAG_Asn150TAG plasmid. Single colonies of freshly transformed cells were cultured in 5 mL of LB media containing 50 μg mL^−1^ kanamycin and 25 μg mL^−1^ chloramphenicol for 18 h at 30 °C. Expression cultures were grown in 96-deepwell blocks sealed with a breathable membrane. 20 μL of the starter culture was used to inoculate 480 μL of defined auto-induction medium containing 50 μg mL^−1^ kanamycin and 25 μg mL^−1^ chloramphenicol (for the cultures with Asn40TAG_Asn150TAG plasmid, IPTG was removed from the auto-induction medium and added when the cultures reached OD_600_ = 0.6). Expression cultures were grown in the presence of the appropriate ncAA (0–10 mM) and incubated at 30 °C with shaking at 850 rpm for 20 h. OD_600_ and GFP fluorescence (*λ*_excitation_: 395 nm, *λ*_emission_: 509 nm) measurements were recorded using a BMG LabTech CLARIOstar spectrophotometer.

### Protein production and purification of MeHis-containing proteins

For the expression of APX2_MeHis, chemically competent *E. coli* BL21(DE3) containing either pEVOL_*Ma*PylRS^IFGFF^/*Ma*^Pyl^tRNA_CUA_ or pEVOL_G1PylRS^MIFAF^/G1^Pyl^tRNA_CUA_ were transformed with pET29b_APX_MeHis. A single colony of freshly transformed cells were cultured in 5 mL of LB media containing 50 μg mL^−1^ kanamycin and 25 μg mL^−1^ chloramphenicol for 18 h at 30 °C. 300 μL of the starter cultures was used to inoculate 30 mL 2xYT medium supplemented with 50 μg mL^−1^ kanamycin, 25 μg mL^−1^ chloramphenicol, 5-aminolevulinic acid (1 mM final) and MeHis (1–0.1 mM final) and cultures were grown at 37 °C, 200 rpm to an OD_600_ of 0.6. Protein expression was induced with the addition of IPTG (0.1 mM final) and arabinose (5 mM final) and the cultures grown for a further 20 h at 20 °C.

For the expression of BH_MeHis_1.8 and OE1.4, chemically competent *E. coli* DH10β containing either pEVOL_*Ma*PylRS^IFGFF^/*Ma*^Pyl^tRNA_CUA_ or pEVOL_G1PylRS^MIFAF^/G1^Pyl^tRNA_CUA_ were transformed with either pBbE8K_ BH_MeHis_1.8 or pBbE8K_OE1.4. A single colony of freshly transformed cells were cultured in 5 mL of LB media containing 50 μg mL^−1^ kanamycin and 25 μg mL^−1^ chloramphenicol for 18 h at 30 °C. 300 μL of the starter cultures was used to inoculate 30 mL 2xYT medium supplemented with 50 μg mL^−1^ kanamycin, 25 μg mL^−1^ chloramphenicol, and MeHis (1–0.1 mM final) and cultures were grown at 37 °C, 200 rpm to an OD_600_ of 0.6. Protein expression was induced with the arabinose (10 mM final) and the cultures grown for a further 20 h at 20 °C.

The cells were harvested and purified as stated above for PylRS purification. Purified proteins were desalted using 10DG desalting columns (Bio-Rad) with PBS pH 7.4 and analysed by SDS-PAGE and protein MS. Protein concentrations were determined by measuring the absorbance at 280 nm using calculated extinction coefficients (ExPASy ProtParam).

### MS analysis

Purified protein samples were diluted to a final concentration of 0.5 mg mL^−1^ with 0.1% acetic acid. MS analysis was performed using a 1200 series Agilent LC, 5 μL injection into 5% acetonitrile (with 0.1% formic acid) and desalted inline for 1 min. Protein was eluted over 1 min using 95% acetonitrile and 5% water. The resulting multiply charged spectrum was analyzed using an Agilent QTOF 6510 and deconvoluted using Agilent MassHunter software.

## Author contributions

A. E. H. and J. F. carried out all laboratory work. J. E. J. S. identified homologs to test and created plasmids for these. All authors discussed the results and participated in writing the manuscript. A. P. G. initiated and directed the research.

## Conflicts of interest

There are no conflicts to declare.

## Supplementary Material

FD-252-D4FD00019F-s001
