# Supplementary material for: An efficient pyrrolysyl-tRNA synthetase for economical production of MeHis-containing enzymes
Source: Faraday Discuss. 2024 Mar 7;252:295–305. doi: 10.1039/d4fd00019f (PMC11389853; doi:10.1039/d4fd00019f)
Supplement: FD-252-D4FD00019F-s001 [file FD-252-D4FD00019F-s001.pdf]

Electronic Supplementary Material (ESI) for Faraday Discussions.
This journal is © The Royal Society of Chemistry 2024

Supplementary Information

An Efficient Pyrrolysyl-tRNA Synthetase for Economical Production of MeHis-containing Enzymes

Amy E. Hutton[1]†, Jake Foster[1]†, James E.J. Sanders[1], Christopher J. Taylor[1], Stefan A. Hoffmann[1], Yizhi Cai[1], Sarah L. Lovelock[1*], Anthony P. Green[1*]

Manchester Institute of Biotechnology, School of Chemistry, The University of Manchester, Manchester, UK.

*Corresponding author: Anthony.green@manchester.ac.uk

Table of Contents

GFP expression data..3

Mass Spectrometry data……5

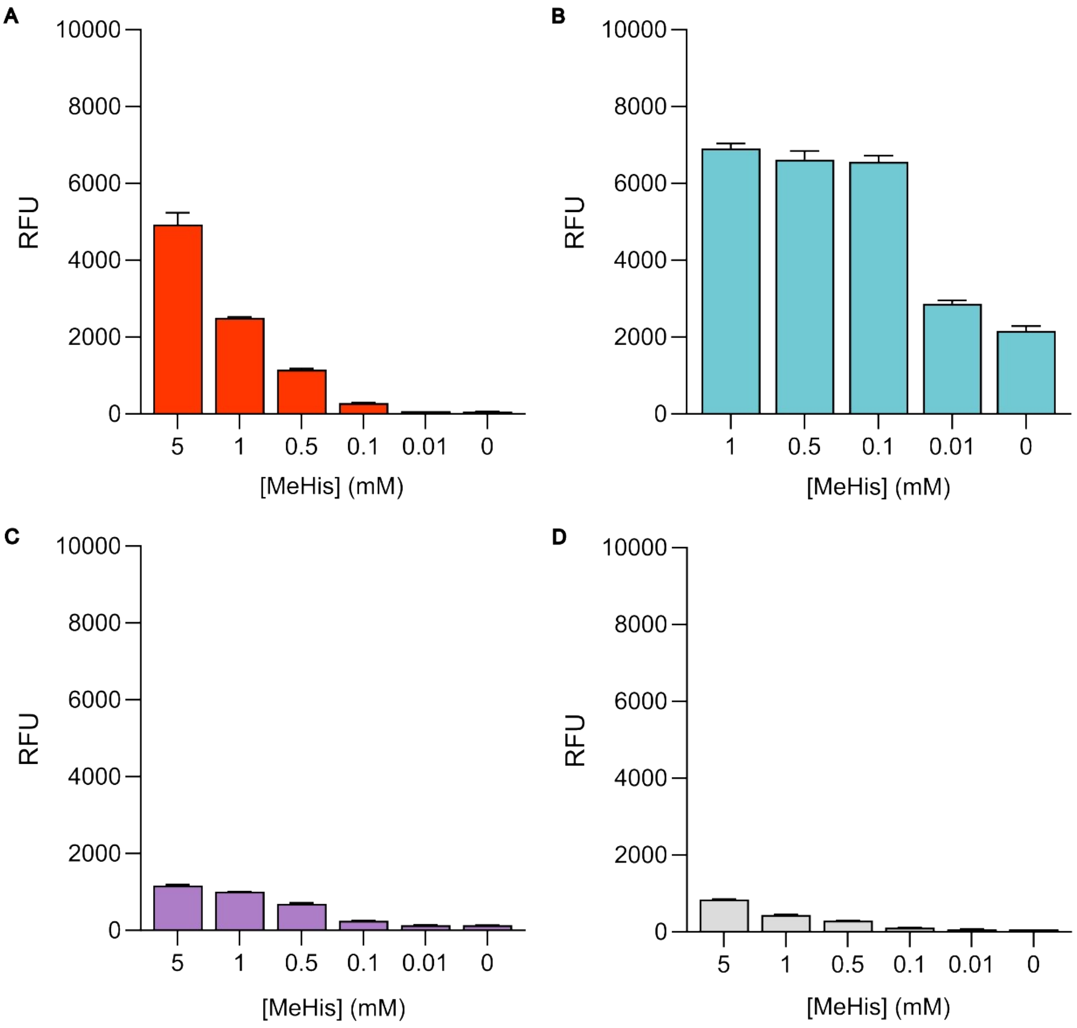

Supplementary Figure 1: Bar charts showing GFP production containing MeHis at position 150 in cultures with varying MeHis concentrations (0-5 mM), using either **A)** *Ma*PylRS^{MIFAF}**; B)** *G1*PylRS^{IFGFF}; **C)** *RumEn*PylRS^{IFGFF}; **D)** *RumEn*PylRS^{MIFAF}. Error bars represent the standard deviation of measurements made in triplicate.

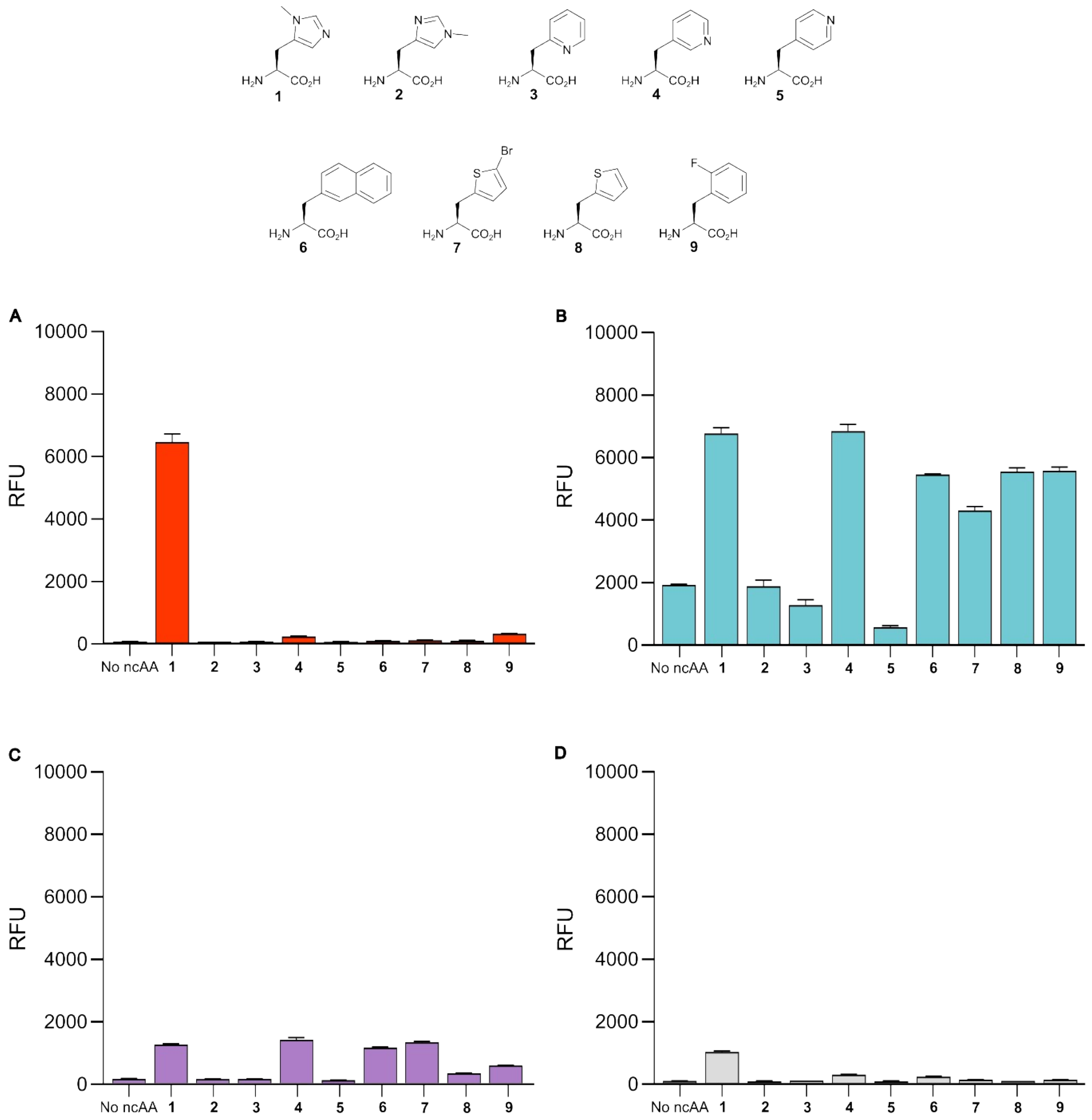

Supplementary Figure 2: Bar charts showing the activities of **A)** *Ma*PylRS^{MIFAF}**; B)** *G1*PylRS^{IFGFF}; **C)** *RumEn*PylRS^{IFGFF}; **D)** *RumEn*PylRS^{MIFAF}, towards a small panel of ncAAs (10 mM, **1-9**) for the production of GFP through the suppression of the UAG codon at position 150. Error bars represent the standard deviation of measurements made in triplicate.

Table S1: Mass spectrometry of GFP variants.

	PylRS	ncAA used	GFP construct	Predicted	Observed
1	*G1*PylRS^{IFGFF}	MeHis	GFP 150UAG (6His-tagged)	27864	27864
2	*G1*PylRS^{MIFAF}	MeHis	GFP 150UAG (6His-tagged)	27864	27864
3	*RumEn*PylRS^{IFGFF}	MeHis	GFP 150UAG (6His-tagged)	27864	27864
4	*RumEn*PylRS^{MIFAF}	MeHis	GFP 150UAG (6His-tagged)	27864	27864
5	*G1*PylRS^{IFGFF}	No ncAA	GFP 150UAG (6His-tagged)	-	27860[a]
6	*G1*PylRS^{MIFAF}	MeHis	GFP 40 and 150UAG (6His-tagged)	27901	27901
7	*G1*PylRS^{MIFAF}	**4**	GFP 150UAG (6His-tagged)	27861	27861
8	*G1*PylRS^{MIFAF}	**6**	GFP 150UAG (6His-tagged)	27946	27946
9	*G1*PylRS^{MIFAF}	**7**	GFP 150UAG (6His-tagged)	27945	27945
10	*G1*PylRS^{MIFAF}	**8**	GFP 150UAG (6His-tagged)	27866	27866

[a] observed mass corresponds to incorporation of phenylalanine.
